# Discriminating high-risk cervical Human Papilloma Virus infections with urinary biomarkers via non-targeted GC-MS-based metabolomics

**DOI:** 10.1371/journal.pone.0209936

**Published:** 2018-12-28

**Authors:** Filipa Godoy-Vitorino, Gilmary Ortiz-Morales, Josefina Romaguera, Maria M. Sanchez, Magaly Martinez-Ferrer, Natalyia Chorna

**Affiliations:** 1 UPR School of Medicine, Department of Microbiology & Medical Zoology, San Juan, Puerto Rico; 2 Inter American University of Puerto Rico, Metro Campus, San Juan, Puerto Rico; 3 UPR School of Medicine, Department of Ob-Gyn, San Juan, Puerto Rico; 4 University of Puerto Rico Comprehensive Cancer Center, San Juan, Puerto Rico; 5 UPR School of Pharmacy, Department of Pharmaceutical Sciences, San Juan, Puerto Rico; 6 UPR School of Medicine, Department of Biochemistry, San Juan, Puerto Rico; Bharathidasan University, INDIA

## Abstract

Genital human papillomavirus (HPV) is the world’s most commonly diagnosed sexually transmitted infection, and high-risk HPV types are strongly linked to cervical dysplasia and carcinoma. Puerto Ricans are among the US citizens with higher HPV prevalence and lower screening rates and access to treatment. This bleak statistic was as a motivation to detect biomarkers for early diagnosis of HPV in this population. We collected both urine and cervical swabs from 43 patients attending San Juan Clinics. Cervical swabs were used for genomic DNA extractions and HPV genotyping with the HPV SPF10-LiPA25 kit, and gas chromatography-mass spectrometry (GC-MS) was employed on the urine-derived products for metabolomics analyses. We aimed at discriminating between patients with different HPV categories: HPV negative (HPV-), HPV positive with simultaneous low and high-risk infections (HPV+B) and HPV positive exclusively high-risk (HPV+H). We found that the metabolome of HPV+B is closer to HPV- than to HPV+H supporting evidence that suggests HPV co-infections may be antagonistic due to viral interference leading to a lower propensity for cervical cancer development. In contrast, metabolites of patients with HPV+H were significantly different from those that were HPV-. We identified three urinary metabolites 5-Oxoprolinate, Erythronic acid and N-Acetylaspartic acid that discriminate HPV+H cases from negative controls. These metabolites are known to be involved in a variety of biochemical processes related to energy and metabolism and may likely be biomarkers for HPV high-risk cervical infection. However, further validation should follow using a larger patient cohort and diverse populations to confirm our finding.

## Introduction

The association between mucosotropic Human Papilloma Virus (HPV) infections and cervical cancer is unequivocal, with the virus being considered the etiologic agent for cervical carcinoma [1, 2]. To date, despite that 180 different HPV types have been sequenced, only about 50 strains infect the epithelium of the genital tract where they can cause cancer or persist asymptomatically [3, 4]. The high-risk HPV types (oncogenic) includes genotypes 16, 18, 31, 33, 35, 39, 45, 51, 52, and 58 that are associated with cervical, vulvar, vaginal, and anal cancer progression, while low-risk types such as 6, 11, 40, 42, 43, 44, 53, 54 and 74 are associated with warts and low-grade anal lesions [5]. Puerto Rico is the region with the lowest cervical cancer screening rates in the US, which has also substantial economic and access barriers to preventive strategies and treatments [6], and a higher prevalence of HPV than compared to those in the US (34% vs 27%) [7, 8].

Screening practices include HPV testing in conjunction with cervical cytology (Pap smear) during a pelvic examination especially for women above the age of 30 years [9]. This test has shown to reduce the incidence of mortality from cervical cancer [10]. However, the test has many barriers that include embarrassment in the screening method that can also contribute to low screening rates in certain cultures such as in Puerto Rico, where screening is significantly low [6]. Therefore, there is a definite need for alternative and supplementary HPV-related infections early detection tests that some authors have discussed before [11]. As an alternative, we aimed to determine if the analysis of urinary metabolites, could be a reliable approach for the screening of patients with cervical HPV infections since the urine is widely used for identification of metabolic biomarkers in cancer [12, 13]. Moreover, as the collection of urine is non-invasive, the biomaterial is very abundant and has a relatively stable composition of proteins—often used to detect prostate and bladder cancer or even relate to inflammatory bowel disease in children [13–15]. Indeed, methods such as HPV DNA urine testing has been used to identify abnormal cells in adolescent girls who do not wish to have a vaginal examination [16, 17]. In fact, it was reported that self-collected urine can be used for HPV DNA detection matching perfectly with the HPV DNA types identified in the corresponding cervical scrapes [18, 19]. This supports our hypothesis that the metabolic changes observed in urine samples could be directly related to the type of cervical HPV infection. Thus, we aimed to determine if changes in urinary metabolites, could be an alternative and reliable approach for the screening of patients with cervical HPV infections.

Urine biofluid samples, to our knowledge, have not been used in conjunction with metabolomics to discriminate patients with cervical HPV infections. However, a recent study suggested that methylation of both host and viral genes in urine has been feasible for cervical cancer screening [20]. Such recent evidence has suggested that biomarkers for cervical cancer may be washed such as exfoliated cells and debris in the urine, a kind of liquid biopsy–that could facilitate the diagnostics of non-urothelial malignant cells such as cervical cancer [21].

In the last decade, there has been an increased trend in the use of “omics” approaches to study cancer biology [22–25]. Among these approaches, metabolomics has been shown to potentially identify relevant biomarkers for cancer detection or for the development of new drug targets. Mass spectrometry-based metabolomics techniques are being used to uncover metabolites in different cancer types [23, 24, 26] due to their reliability and reproducibility [27].

Taken together these evidence supports our study, aimed at testing if urine could be used as a non-invasive method for the detection of cervical HPV infections by evaluating the association between cervical HPV types and urinary metabolites.

## Materials and methods

### Patient recruitment and sampling

Women undergoing gynecology evaluation at the University of Puerto Rico and San Juan City clinics (San Juan Metropolitan area), who did not meet the exclusion criteria, were recruited to the study. The exclusion criteria were: 1) antibiotics taken in the prior 2 months; 2) history of regular urinary incontinence; 3) treatment for or suspicion of prior toxic shock syndrome; 4) candidiasis; 5) active urinary tract infections; 6) active STDs; and 7) vaginal irritation at the time of screening. The study was approved by the Ethics Committees of the UPR-Medical Sciences Campus IRB (Protocol ref. 1050114/June 2014), San Juan City Hospital and the Inter American University of Puerto Rico IRB (Protocol ref. 1182327–2014) as part of a larger cervical microbiome study. All subjects were informed (both verbally and in writing) of the sampling procedure, risks and benefits of the study, gave written informed consent and signed HIPAA forms, in accordance with the Declaration of Helsinki.

Urine biofluid was self-collected at the time of gynecology evaluation, from 43 healthy reproductive-age women (21–50 years old), with the ability to provide informed consent. Metadata categories we collected from the interview/visit included age, BMI and smoking (S1 Table). All samples were stored at -80°C and processed for further metabolite extraction. Additionally, conventional cervical cytological test (Pap smear) was obtained for cytology diagnostics. Cervical swabs were obtained from the patients using sterile Catch-All Specimen Collection Swabs (Epicentre Biotechnologies, Madison WI), and placed in MoBio bead tubes with buffer (MoBio PowerSoil^™^ kit, MoBio, Carlsbad CA) for genomic DNA extractions using the MoBio PowerSoil^™^ kit, following the manufacturer’s instructions. As these patients were recruited as part of a cervical microbiome study, the cervical genomic DNA extractions used both for HPV typing and microbiota analyses, were done using the standard MoBio soil kit as suggested by the Manual of Procedures of the Human Microbiome Project protocol [28].

### Human Papilloma Virus genotyping

For HPV genotyping we used a short-polymerase chain reaction-fragment assay (Labo Bio-medical Products (LBP), Rijswijk, The Netherlands, licensed Innogenetics technology) using the cervical genomic DNA. The assay uses SPF10 primers to amplify a 65-bp fragment of the L1 open reading frame of HPV genotypes, followed by a Reverse-Hybridization step. In the first step, the 65-bp PCR fragment assay amplifies HPV genotypes: 6, 11, 16, 18, 31, 33, 34, 35, 39, 40, 42, 43, 44, 45, 51, 52, 53, 54, 56, 58, 59, 66, 68/73, 70, and 74. In the second step, the amplified fragments underwent a line probe assay by reverse-hybridization, to determine the specific HPV type compared to the kit-provided controls [29].

### Metabolomics procedure and analysis

#### Metabolite extraction and derivatization

Two hundred μL of liquefied urine samples were mixed with 800 μL of the methanol-water mixture (8:1 v/v), vortexed for 1 min and centrifuged at 13000 rpm X 10 min at 4°C. After centrifugation, supernatants were collected, placed in glass vials, and evaporated to dryness using a SpeedVac (Savant AS160, Farmingdale, NY). The metabolite samples were first derivatized by methoxyamination by adding 50 μL of 20 mg/mL solution of methoxyamine hydrochloride (Sigma-Aldrich) in pyridine (Sigma-Aldrich) and incubated at 37°C for two hours. Trimethylsilylation was subsequently performed by adding 50 μL of N-methyl-N-trimethylsilyl-trifluoroacetamide (MSTFA+1% TMCS, Sigma-Aldrich), incubating for 1 h at 65°C and centrifuged at 13000 rpm X 10 min at RT. Supernatants were transferred to analytical vials and stored at -20°C or diluted in hexane (1:50) prior to the GC-MS analysis.

#### Metabolites separation by GC and detection by MS

Metabolites were fractionated by gas chromatography-mass spectrometry (GC-MS) (GCMS-QP2010, Shimadzu Scientific). The chromatography conditions were as follows: RXI-5MS (0.25 mm inner diameter, 0.25 μm D.F., 30 m) (Restek), split injection (ratio = 15), the injection volume of 1 μL. The inlet temperature was 280 °C; the ion source temperature was 200 °C; interface temperature was 150 °C. The oven temperature was set at 100 °C for 1 min, and then programmed from 100 °C to 290 °C at 8 °C/min, and held at 290 °C for 16 min. Helium was used as the carrier gas at a constant linear velocity of 39 cm/sec. The sample aliquot was injected in split mode (ratio = 15). Mass spectra were obtained for each metabolite on a Shimadzu GCMS-QP2010 mass spectrometer after electron impact ionization (EI, 70 eV, ion source temperature 200°C) in full scan mode between 35 and 700 amu. Mass spectral library searches of the major chromatographic peaks were conducted using the GCMS Labsolution data analysis software (Shimadzu) equipped with NIST14/2014/EPA/NIH database.

#### Bioinformatics analysis

Genotyping results were grouped into three main categories: HPV negative (HPV-), HPV positive with simultaneous low and high-risk infections (HPV+B), and HPV positive exclusively with high-risk genotypes (HPV+H). Studies had found multiple HPV genotypes associated with neoplasias [30], including low-risk types [31]. Recently HPV research has also grouped patients with both high-risk and low-risk infections in studies relating HPV diversity and prevalence in Hispanic populations [32, 33].

For metabolomics analysis, peak intensities for each metabolite were collected, composed as the data matrix and processed using Metaboanalyst 4.0 [34, 35] unless otherwise specified. Data integrity check was performed according to default settings on the Metaboanalyst interface. Thus, obtained datasets were evaluated by Principal Component Analysis to identify samples-outliers being outside the Hotelling T^2^ 95% confidence ellipse [36]. Next, identified outliers (two in HPV+B and one HPV+H group) and samples that did not produce a total ion chromatogram (Table 1) were removed from further analysis. The resultant data were normalized by log-transformation and Pareto scaling to improve the pattern recognition for untargeted metabolomics data. Changes between groups were analyzed via the Holm-Sidak test for multiple comparisons with GraphPad Prism version 7.0c (GraphPad Software, San Diego, CA). The α level for significance was set at 0.05. The supervised Partial Least-Squares Discriminant Analysis (PLS-DA) model was used to identify the metabolic differences between groups. To evaluate the model performance, class labels were permuted 2,000 times to identify whether differences between groups were significant. For permutation test statistics we selected separation distance, which was defined as the ratio of the between-group sum of squares and the within-group sum of squares (B/W ratio). Group’s performance was evaluated by using the leave-one-out cross-validation (LOOCV) method. Evaluation of Variable Importance in Projection (VIP) scores, estimated the importance of each variable in the projection used within the PLS-DA model. Variables with a VIP score ≥ 1.0 were considered important in a given model. The diagnostic ability of these variables as potential biomarkers for the detection of HPV infections in urine, was assessed by measuring the area under the curve values (AUC), using the receiver operating characteristic curves (ROC) method. Variables with a AUC ≥ 0.9 were considered important for diagnostic purposes [37].

**Table 1:** Sample IDs and groupings by HPV-risk, genotypes and cytology.

#	Sample ID	Genotyping Result	Cytology	HPV Risk	HPV Status	Group
1	35	Negative	LGSIL	Negative	Negative	HPV-
2	50	Negative	ASCUS	Negative	Negative	HPV-
3	53**	Negative	HGSIL	Negative	Negative	EXC
4	58	Negative	LGSIL	Negative	Negative	HPV-
5	61	Negative	HGSIL	Negative	Negative	HPV-
6	69	Negative	HGSIL	Negative	Negative	HPV-
7	70	Negative	HGSIL	Negative	Negative	HPV-
8	75	Negative	HGSIL	Negative	Negative	HPV-
9	79	Negative	NSIL	Negative	Negative	HPV-
10	16	16,66,6	LGSIL	Both	Positive	HPV+B
11	18*	16,6,53	HGSIL	Both	Positive	EXC
12	21	31,33,42,44,74	HGSIL	Both	Positive	HPV+B
13	22	16,31,39,45,66,68,44,53,74	LGSIL	Both	Positive	HPV+B
14	25*	16,33,66,6	HGSIL	Both	Positive	EXC
15	26	16,51,56,6,34,44,53,74	LGSIL	Both	Positive	HPV+B
16	27	39,74	LGSIL	Both	Positive	HPV+B
17	28	54,56	HGSIL	Both	Positive	HPV+B
18	30	16,39,52,53,56	HGSIL	Both	Positive	HPV+B
19	31**	31,52,6,74	LGSIL	Both	Positive	EXC
20	34	16,56,74	HGSIL	Both	Positive	HPV+B
21	36	18,44,74	HGSIL	Both	Positive	HPV+B
22	47	31,33,44	HGSIL	Both	Positive	HPV+B
23	48	33,42	ASCUS	Both	Positive	HPV+B
24	49	33,42	HGSIL	Both	Positive	HPV+B
25	51	51,53	ASCUS	Both	Positive	HPV+B
26	63	51,53,66	HGSIL	Both	Positive	HPV+B
27	65	54,45,51	NA	Both	Positive	HPV+B
28	66	31,6	HGSIL	Both	Positive	HPV+B
29	17	16,66	HGSIL	H-risk	Positive	HPV+H
30	19	35	HGSIL	H-risk	Positive	HPV+H
31	20	66	LGSIL	H-risk	Positive	HPV+H
32	32	52	LGSIL	H-risk	Positive	HPV+H
33	33	16	HGSIL	H-risk	Positive	HPV+H
34	37	31	LGSIL	H-risk	Positive	HPV+H
35	43	51	LGSIL	H-risk	Positive	HPV+H
36	44*	56	LGSIL	H-risk	Positive	EXC
37	56	18,35	ASCUS	H-risk	Positive	HPV+H
38	60	51,52,66	LGSIL	H-risk	Positive	HPV+H
39	62	16	LGSIL	H-risk	Positive	HPV+H
40	68	68	HGSIL	H-risk	Positive	HPV+H
41	72**	45	HGSIL	H-risk	Positive	EXC
42	74**	31	NA	H-risk	Positive	EXC
43	78**	52	ASCUS	H-risk	Positive	EXC

*—outlier removed from metabolomics analysis;

**—sample did not produce the total ion chromatogram; EXC—samples excluded from the metabolomics analysis.

Cytology categories: NA-undetermined; LGSIL Low-grade squamous intraepithelial lesion, HGSIL high-grade squamous intraepithelial lesion, ASCUS Atypical squamous cells of undetermined significance, and NSIL Negative for squamous intraepithelial lesion.

## Results and discussion

Cervical HPV genotyping of the 43 patients revealed a total of 34 HPV positive patients and 9 negative to HPV infections (HPV-). Of the 34 HPV positives, 15 were exclusively high-risk types (HPV+H) and 19 had simultaneously high-risk and low-risk infections, ranging from 2 to 9 simultaneous HPV genotypes (HPV+B) (Table 1). We did not find any patients positive only for low-risk HPV types. These 43 patients were mostly non-smokers, and although socio-demographic and cytology metadata information was available, due to the modest sample size these categories did not pass the PLS-DA model validation.

The distribution of multiple HPV infections is common but with different HPV co-infection prevalence rates in different countries. Thus, it was documented that out of the 2,478 samples from the Costa Rican HPV Vaccine Trial, 43.2% had multiple HPV type infections [38]. In a study of 5,000 samples from the Centralized Cervical Cancer Screening Program of British Columbia, 33% were positive for more than one HPV type [39]. In Brazil, a study recruiting 2,113 women for a 1-yearand a 4-year period, showed an HPV co-infection prevalence of 12.3% and 22.3% correspondingly [40]. Relatively similar to the co-infection prevalence in Costa Rica, our study found 44.2% of Puerto Rican woman positive for multiple HPV type infections (Table 1). Other studies performed in Venezuela, revealed Amerindian populations with a 75% HPV prevalence, and with 23 different HPV types, a study that discriminated between exclusively low-risk HPV, exclusively high-risk and co-infections by both HPV types [32], as well as another study that made grouping between low-risk and high-risk types [33].

A total of twenty-four metabolites were identified in samples from all groups according to their electron impact mass spectra by comparison to the NIST14 spectral mass library (Table 2).

**Table 2:** Human urine metabolome found in HPV+B vs HPV- and HPV+H vs HPV- groups.

#	Metabolite	RT	Fragment ions	VIP	HPV+B Adj P	HPV+H Adj P
1	Lactic acid	9.67	189, 233, 261	0.47	1.000	0.988
2	Acetic Acid	9.90	189, 219, 247	1.11	1.000	0.599
3	Glycine	10.69	147, 218, 246	0.47	1.000	0.993
4	2-Hydroxybutyric acid	11.24	189, 247, 275	0.55	1.000	0.988
5	Proline	11.55	184, 258, 328	0.22	0.987	0.993
6	Methylmalonic acid	11.83	147, 189, 289,	0.61	1.000	0.988
7	Urea	12.09	147, 231, 273	0.20	1.000	0.993
8	**5-Oxoprolinate**	13.18	75, 158, 186	1.82	0.303	0.009*
9	Threonine	13.27	130, 246, 290	0.61	1.000	0.983
10	Succinic acid	13.47	147, 189, 289,	0.57	1.000	0.955
11	Glycerol	15.07	171, 189, 347	0.35	1.000	0.993
12	5-Oxoproline	15.97	147, 272, 300	1.23	1.000	0.205
13	Glutaric acid	16.13	73, 147, 303	1.20	1.000	0.365
14	**N-Acetylaspartic acid**	16.65	73, 147, 346	1.65	0.575	0.024*
15	2-Butenoic acid	17.11	189, 247, 275	0.45	1.000	0.988
16	Hippuric acid	17.64	77, 105, 236	0.73	1.000	0.983
17	**4-Hydroxybutyric acid**	17.72	73, 147, 275	1.62	1.000	0.035*
18	4-Hydroxyphenylacetic acid	17.83	75, 205, 324	0.68	1.000	0.955
19	**Erythronic acid**	18.28	147, 289, 331	1.69	1.000	0.016*
20	2-Hydroxyglutaric acid	19.04	147, 207, 433	0.32	1.000	0.993
21	Glyceric acid	19.95	73, 231, 391	0.04	1.000	0.993
22	Aconitic acid	20.8	73, 147, 459	1.46	1.000	0.120
23	Citric acid	23.14	357, 459, 591	1.27	0.909	0.599
24	Uric acid	25.47	73, 567	1.11	1.000	0.685

RT—Retention time; VIP—Variable importance in projection scores identified via PLS-DA analysis for the PC1; Adj P–Adjusted P value determined using the Holm-Sidak method in comparison to HPV- group, α = 0.05.

*—indicate metabolite that matches selected criterion.

**Fig 1:**
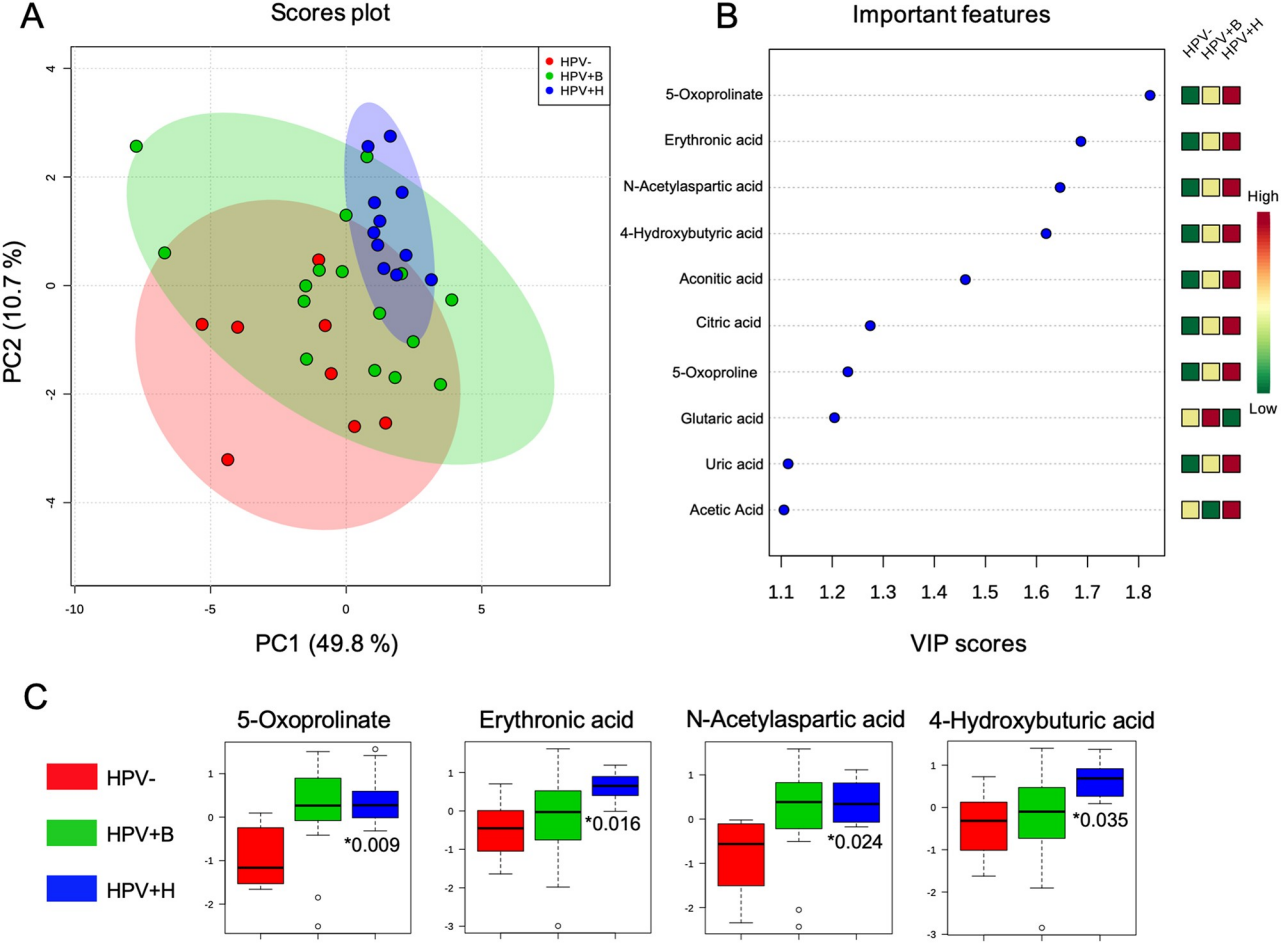
Metabolites discriminating HPV-, HPV+B and HPV+H patients. (A) Partial-least discriminant analysis (PLS-DA) score plot. (B) Variable importance in projection (VIP) plot with cut-off ≥ 1.0. (C) Box-and-whisker plots depict the most significant metabolites, with the top two: 5- Oxoprolinate and N-Acetylaspartic acid changes in HPV+B and HPV+H groups relative to HPV-. *Adjusted P value. Y-axis shows the normalized relative abundance. RT—Retention time; VIP—Variable importance in projection scores identified via PLS-DA analysis for the PC1; Adj P–Adjusted P value determined using the Holm-Sidak method in comparison to HPV- group, α = 0.05. *—indicate metabolite that matches selected criterion.

To identify potential biomarkers discriminating between patients with HPV infections and those that had no infections, we performed PLS-DA analysis using the normalized metabolite intensities as variables. The PLS-DA was used to analyze three groups of patients. PLS-DA scores plot of HPV- and HPV+H displayed satisfactory separation at the 95% level with minor overlap between source ellipses, while the separation between HPV- and HPV+B was less significant suggesting similarity in the metabolites abundances between HPV- and HPV+B. The PLS-DA showed that 60.5% of the total explained variance in the data was represented by the first two principal components (PC1–49.8% and PC2–10.7%). The permutation test showed significant separation distance between groups (p < 5e-04) (Fig 1A).

This suggests that in our study, the metabolome of HPV+B group is closer to HPV- than to HPV+H. Thus, it is very likely that the pattern of HPV genotype co-infections in HPV+B group does exhibit a rather antagonistic effect of the different HPV risk types, resulting in a similar metabolomic profile as in HPV-. The effects of multiple interactions between co-infecting HPV types on carcinogenesis are not well studied and data reported to date are controversial. Some reports suggested that multiple HPV infections could trigger synergistic effects enhancing the development of carcinogenesis [41, 42] while others reported their antagonistic interactions, that likely may reduce the risk of cervical cancer [43–45]. In addition, despite the popular opinion that multiple HPV infections are associated with the higher risk of cervical cancer compared to those with single HPV infection, several studies conducted in different counties identified that multiple HPV infections are not necessarily correlated with the severity of cervical abnormalities [5, 46, 47]. Therefore, there is a need to perform more studies evaluating the incidence of multiple type HPV infections in different populations, and identification of any interaction between HPV types in the incidence of cervical cancer.

We next identified ten significant discriminatory metabolites (VIP score ≥1) responsible for the separation of groups that may have a clinical value in the diagnosis of HPV infection. Thus, we found higher abundance of 5-Oxoprolinate (VIP, 1.8), Erythronic acid (VIP, 1.7), N-Acetylaspartic acid (VIP, 1.6), 4-Hydroxybuturic acid (VIP, 1.6), Aconitic acid (VIP, 1.5), Citric acid (VIP,1.3), 5-Oxoproline (VIP, 1.2), Glutaric acid (VIP, 1.2), Uric acid (VIP, 1.1) and Acetic acid (VIP, 1.1) (Table 2, Fig 1B). These metabolites are known to be involved in multiple biochemical processes, such as energy metabolism, pentose phosphate pathway, γ-glutamyl cycle and futile 5-Oxoproline cycle.

To determine whether these metabolic changes were significant, we performed the Holm-Sidak corrections for multiple comparisons (α = 0.05) using the following group combinations HPV- with HPV+B, HPV- with HPV+H and HPV+B with HPV+H. We found that 5-Oxoprolinate, Erythronic acid, N-Acetylaspartic acid, and 4-Hydroxybuturic acid were significantly elevated in HPV+H group compare to HPV- (Table 2, Fig 1C). As expected, we have not identified significant changes in HPV+B vs HPV-, since the metabolome of HPV+B group is closer to HPV- than to HPV+H. However, multiple test comparisons have not identified changes between HPV+B vs HPV+H. The biological significance of this is to be determined, and requires a using a larger patient cohort.

**Fig 2:**
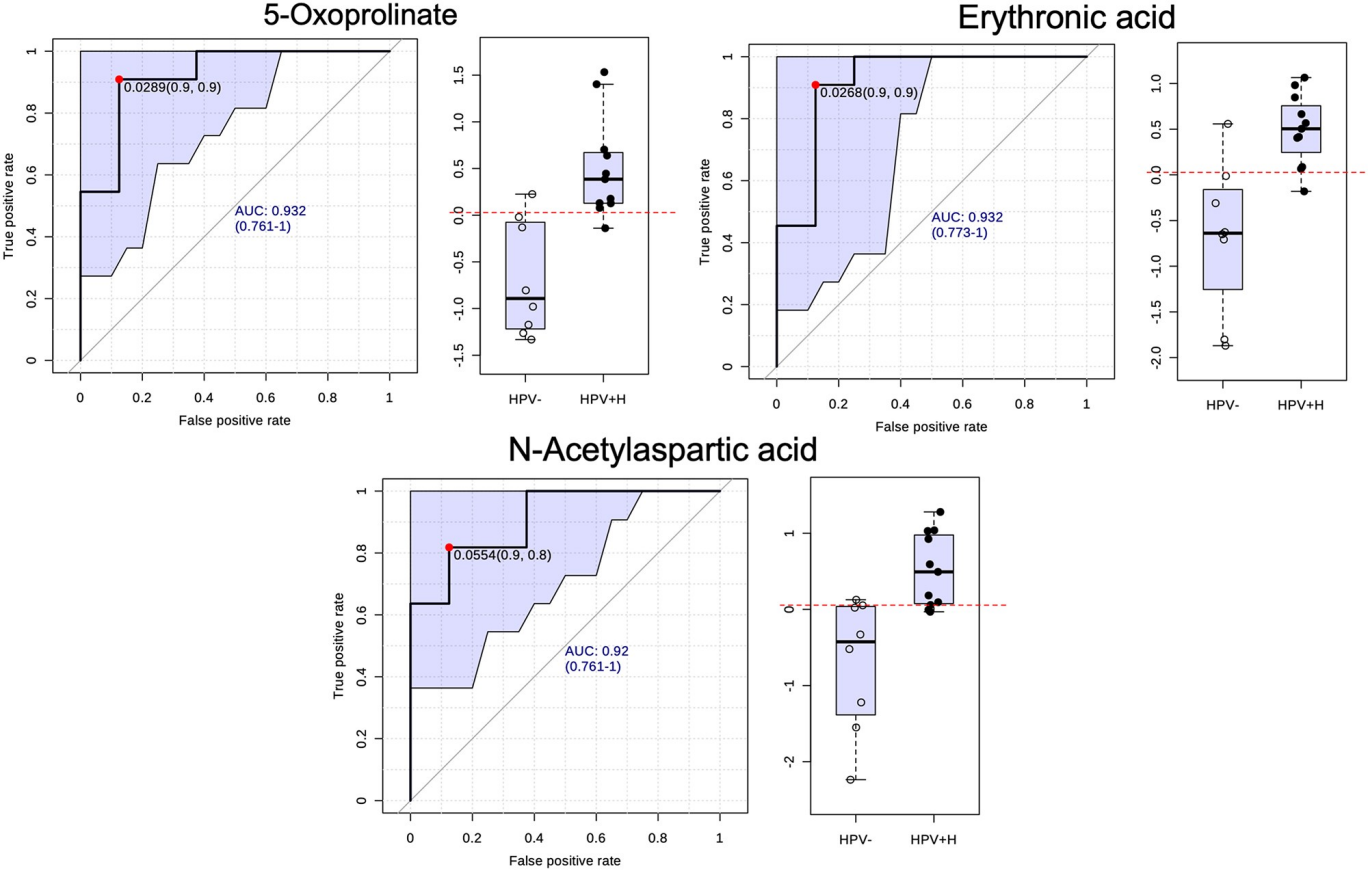
Urine biomarkers predicted by ROC curve analysis curve with 95% confidence interval (shadowed). Box-and-whisker plots show the distribution of abundance values of 5-Oxoprolinate, Erythronic acid and N-Acetylaspartic acid in HPV- vs HPV+H samples with the optimal cut-off as a horizontal dotted red line.

To identify the diagnostic potential of the four most significant metabolites 5-Oxoprolinate, Erythronic acid, N-Acetylaspartic acid and 4-Hydroxybuturic acid as prognostic biomarkers for high-risk HPV infection, we conducted an additional ROC analysis (Fig 2). The analysis showed the greatest AUC values in HPV+H vs HPV- including 5-Oxoprolinate (AUC, 0.92), Erythronic acid (AUC, 0.92) and N-Acetylaspartic acid (AUC, 0.91). Taken together, 5-Oxoprolinate, Erythronic acid and N-acetylaspartic acid could serve as prognostic biomarkers to discriminate high-risk HPV infections from non-infected controls.

5-Oxoprolinate (the conjugate base of 5-Oxoproline) is an intermediate in the isomerization of glutathione to 5-Oxoproline via the action of γ-glutamyl cyclotransferase in the γ-glutamyl cycle. Elevation of 5-Oxoproline levels in blood and urine has been increasingly recognized as a cause of 5-Oxoprolinuria [48] that usually occurs in chronically ill, malnourished women with impaired renal function and chronic acetaminophen ingestion [49]. In addition, HPV infections in women are usually associated with a low level of glutathione [50], decreased expression of antioxidant enzymes and elevation of ROS levels in host cells [51]. Reduction in glutathione content augments an ATP-depleting futile 5-Oxoproline cycle via elimination of the feedback inhibition of γ-Glutamyl Cysteine Synthetase causing increase of γ-Glutamyl Cysteine that is subsequently metabolized to 5-Oxoproline which could cycle back into glutamic acid via action of 5-Oxoprolinase and at the cost of two ATP molecules without production of Glutathione [49, 52, 53].

Erythronic acid is a normal organic acid present in biofluid samples of healthy children and adults. However, its elevation in urine evidences a deficiency of the activity of Transaldolase—a key enzyme in the pentose phosphate pathway [54]. This can lead to various clinical manifestations including liver dysfunction hepatosplenomegaly, hepatic fibrosis in the pathophysiology of diabetes [55, 56].

N-Acetylaspartic acid is synthesized from Aspartate and Acetyl-CoA and via hydrolysis of N-Acetyl-aspartylglutamate. According to the literature, elevation of N-Acetylaspartic acid could be regulated by the Ras homolog gene family, member C guanosine triphosphatase, which is essential in transforming growth factor beta 1- induced epithelial-mesenchymal transition in cervical cancer cells [34]. To date, this metabolite is only known as a specific urinary marker for Canavan disease [57] which is caused by Aspartoacylase deficiency with abnormal accumulation of N-acetylaspartic acid in the brain and body fluids [58].

To date, HPV testing is increasingly used for screening in conjunction with the conventional cervical cytological test (Pap smear) or liquid-based cervical cytological test [9, 59] followed with the HPV DNA test [11]. Cervical cytology classification for squamous cell abnormalities includes Atypical Squamous Cells of Undetermined Significance (ASCUS), Negative for squamous intraepithelial lesion (NSIL), Low-Grade, and High-Grade Squamous Intraepithelial Lesion (LGSIL and HGSIL). Given that, these types of cervical screenings are invasive and time-consuming, the development of new diagnostic methods using biofluid samples including urine could provide a feasible alternative to HPV testing of cervical samples [60]. Thus, our study provides a new possibility for the detection of HPV+H infections in urine, via analysis of the content of 5-Oxoprolinate, Erythronic acid and N-Acetylaspartic acid using just 200 μL of the urine sample. The applicability of these metabolites as predictive urinary markers requires further investigation using a larger patient cohort, which is the limitation of the current study. In addition, assessment of Erythronic acid and N-Acetylaspartic acid abundance in urine are already routinely performed, via DNA mutation screenings for Transaldolase and Aspartoacylase correspondently. The existing use of these laboratory tests confirms its suitability for the diagnosis of HPV+H infections. Current diagnosis of 5-Oxoprolinuria is expensive and performed only in specialized laboratories via GC-MS. Nevertheless, this study suggests that mutational analysis of the 5-Oxoprolinase gene may be advisable for routine diagnostic purposes [61] which could also apply for the identification of HPV+H infections in clinical laboratories.

## Conclusions

This study provided preliminary evidence for the successful detection of urine metabolites related to cervical high-risk HPV infections. Using the urine samples of the Puerto Rican woman followed by GC-MS analysis we have shown, that patients with high-risk HPV infections have the significantly higher abundance of 5-Oxoprolinate, Erythronic acid, and N-Acetylaspartic acid. Besides characterizing cervical HPV, we were able to relate high-risk HPV infections with urinary metabolites and defined 5-Oxoprolinate, Erythronic acid, and N-Acetylaspartic acid as possible prognostic biomarkers for high-risk HPV infections. We also found that patients with simultaneous high-risk and low-risk infections had a similar urinary metabolome with patients without infections supporting early evidence that suggests HPV co-infections may be antagonist due to viral interference leading to lower propensity in cervical cancer development. However, further validation should follow using a larger patient’s cohort to confirm our finding. In addition, it is advisable to perform more MS-based studies to evaluate differentially abundant metabolites and peptides in urine that may correlate not only with HPV genotypes but with cervical intraepithelial neoplasia stages and clinical status.

## Supporting information

S1 Table Metadata categories for the 43 patients. (XLSX)
